# Mediterranean dietary pattern analysis combining two nutritional assessment tools in children aged 3 to 6 years in five European countries

**DOI:** 10.1007/s00394-026-03930-y

**Published:** 2026-04-15

**Authors:** Alexander Triebswetter, Julia Erhardt, Martina Totzauer, Verònica Luque, Mariona Gispert-Llaurado, Elvira Verduci, Dariusz Gruszfeld, Annick Xhonneux, Berthold Koletzko, Veit Grote

**Affiliations:** 1 Division of Metabolic and Nutritional Medicine, Department of Paediatrics, Dr. von Hauner Children´s Hospital, LMU University Hospital, Munich, Germany; 2 German Center for Child and Adolescent Health, Munich, Germany; 3 Paediatrics Research Unit, Universitat Rovira I Virgili-IISPV, 43201 Reus, Spain; 4 Serra Hunter Fellow, Universitat Rovira I Virgili-IISPV, 43201 Reus, Spain; 5 Metabolic Disease Unit, Department of Paediatrics, Vittore Buzzi Children’s Hospital, University of Milan, 20154 Milan, Italy; 6 Neonatal Department and Neonatal Intensive Care Unit, Children’s Memorial Health Institute, 04-730 Warsaw, Poland; 7 Groupe Santé CHC, Bd. Patience et Beaujonc 2—(B), 4000 Liège, Belgium

**Keywords:** Dietary pattern, Mediterranean diet, Children, Childhood obesity project, Food diary, Food frequency questionnaire

## Abstract

**Purpose:**

While the Mediterranean diet (MD) is associated with numerous health benefits, in children there is limited information on effects of assessment methods, cross-country differences, and the tracking of MD adherence over time. We aimed to compare and combine dietary assessment tools in young children, to provide an informative method for scoring adherence to MD, and to examine cross-country variations.

**Methods:**

3-Day Food Diaries (3-DFD) and Food Frequency Questionnaires (FFQ) were assessed at 4 time points during the ages of 3 to 6 years across five European countries: Belgium, Germany, Italy, Poland, and Spain. Information from both tools was used to calculate the Mediterranean Diet Quality Index for children and adolescents (KIDMED), both independently and in combination. For the latter, country and time point differences were examined. Individual diet score variance per country across time points was determined using Intraclass Correlation Coefficient (ICC).

**Results:**

The combined KIDMED score (3.76 ± 2.28) was higher compared to both the FFQ (3.31 ± 2.33) and the 3-DFD score (1.78 ± 2.45) across follow-ups. The combined KIDMED score was higher in Mediterranean countries (Italy 5.00 ± 1.60; Spain 4.54 ± 1.70) than non-Mediterranean countries (Belgium 1.96 ± 2.07; Germany 3.13 ± 2.01; Poland 1.65 ± 2.12) (*p* < 0.01), driven by more children from Mediterranean countries consuming *fruits*,* vegetables*,* fish*,* pulses*, and *olive oil*. The combined KIDMED score was rather stable over time. Intra-individual consistency over time was poor to moderate (Germany: 0.620, Belgium: 0.604, Italy: 0.429, Poland: 0.564, and Spain: 0.493).

**Conclusions:**

Combining FFQ and 3-DFD led to a higher KIDMED score reflecting more detected details of frequencies in food consumption. MD adherence was poor to moderate and remained stable over time in early childhood, suggesting that dietary patterns established at a young age are likely to persist.

*Trial registration:* This trial was registered at clinicaltrials.gov as *NCT00338689*.

**Supplementary Information:**

The online version contains supplementary material available at 10.1007/s00394-026-03930-y.

## Introduction

The Mediterranean Diet (MD) describes a traditional dietary pattern of people living in Mediterranean areas, particularly in olive-tree-growing regions [1]. MD is characterized by a high consumption of olive oil, but also fruits, vegetables, whole grains, legumes, nuts, and seeds. Regular but moderate dairy intake (milk, yogurt, cheese), moderate amounts of fish and poultry, minimal consumption of red meat, and moderate amounts of wine during meals are further aspects of the MD [1, 2]. An extensive and consistent body of evidence associates adherence to MD in adults with various health benefits, such as reduced risks of cardiovascular disease and diabetes, as well as lower blood pressure, blood cholesterol, and all-cause mortality [3–8]. Due to its positive reputation, the possible impact of MD on important health outcomes has also been studied in children [9–11]. It was shown that adherence to a MD in children is not only associated with a lower body mass index (BMI) [12–16], but also with reduced systolic blood pressure [13], total cholesterol [14, 16], low-density lipoprotein, and triglyceride [14]. Dietary intakes of ω − 9 fatty acids, selenium, vitamin E, and zinc increase with a MD compared to a Western type diet [14]. MD was also associated with positive effects on inflammatory pathways and oxidative stress [15].

According to a systematic review from 2017, the most widely applied tool to evaluate a child’s diet concerning the MD is the Mediterranean Diet Quality Index for children and adolescents (KIDMED) questionnaire, originally developed in 2003 in Spain by Serra-Majem et al. for subjects aged 2–24 years [9, 17, 18]. Each question represents a specific dietary habit, such as regular consumption of olive oil, fish, pastries, processed meat, and others. Negative and favorable food habits are respectively assigned negative or positive scores, resulting in a KIDMED score with a higher score indicating greater adherence to the MD. In subsequent years this scoring system was applied in other Mediterranean countries, but also in non-Mediterranean regions [19–22] and became therefore a frequently utilized measurement tool in studies. This questionnaire was mostly used directly, but also applied to information from Food Frequency Questionnaires (FFQ) and less often from 24h-recalls or 3-Day Food Diary (3-DFD). For a few studies, the questions (also called “items”) were modified by the authors, to get a more appropriate tool for their research objective, respectively [23–25]. Recently, an updated KIDMED 2.0 version was published (e.g. excluding fruit juice from the fruit categories) and validated via a 7-day dietary record in Spanish children and adolescents [26]. In response to calls to expand knowledge on the MD indices [9], we investigate the impact of different dietary assessment tools and their combination to measure adherence to the KIDMED score. Our aims are to compare these tools, to provide an informative method for scoring adherence to MD, to describe adherence to MD in young children from 5 European countries over time, and to examine cross-country variations used in the European Childhood Obesity Project (CHOP), i.e. 3-DFD and FFQ, on the KIDMED score.

## Methods

### Study design and population

This study is an observational prospective analysis of dietary data secondary to the CHOP trial, conducted in 5 European countries (Germany, Belgium, Italy, Poland, and Spain). CHOP was a double-blind, multicenter, randomized controlled intervention trial in which 1,678 healthy infants were recruited during their first eight weeks of life since 2002. Part of the children were assigned to receive infant formula with different protein contents in the first year of life to investigate effects on growth and later obesity risk; in addition, an observational group of breastfed infants was included as a reference group. The energy difference between the formula groups was compensated by adjusting the fat content of the lower protein formula via a mixture of plant-based oils (coconut, palm, rapeseed, and sunflower oils, emulsified with soy lecithin). While total protein and fat content differed between formulas, the protein and fat composition did not change. Follow-up in subsequent years included diet surveys, blood measurements, mental health surveys, and other tests. Details on methodology, e.g. formula composition, and results of the CHOP study were published elsewhere [27, 28]. The trial was registered in clinicaltrials.gov ID NCT00338689.

The study population used for this analysis was restricted to participants who completed a 3-DFD and a FFQ at least at one of the four follow-up time points (3, 4, 5, and 6 years), independent of assigned intervention group.

### Dietary assessment

Dietary intake was assessed through 3-DFDs and FFQ in children aged 3, 4, 5, and 6 years. 3-DFD were completed by parents or caregivers on three days of the week (2 weekdays, and 1 weekend day) with estimated or weighed food and beverage intakes. These dietary records were entered in a software specifically developed for the CHOP study, with nutrient composition data of food products based on the German food composition database BLS 2.3 (Bundeslebensmittelschlüssel) [29]. Nutrient content of food items was updated with BLS 3.01 for analysis and other national data bases [30–32]. Trained dieticians checked 3-DFD for completeness and accuracy and discussed any issues with parents before data entry following standard operating procedures [33, 34]. The entered individual food and beverage items were assigned to food groups according to their food type and nutrient composition of which 44 major food groups were considered as relevant and were therefore used in the present analysis (Supplementary Table 1).

**Fig. 1:**
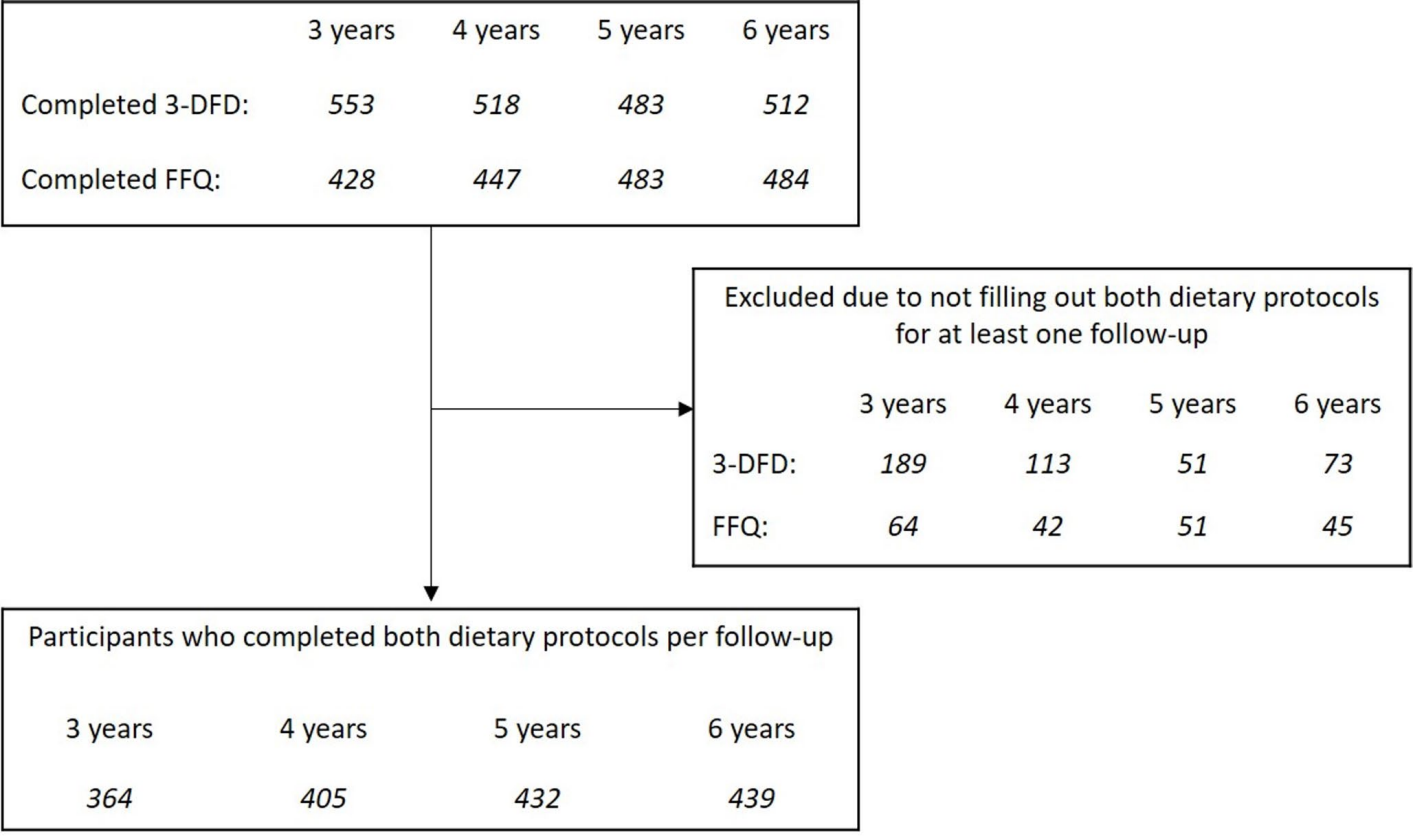
Flow chart of selection of study population based on availability of 3-day food diaries and food frequency questionnaires at age 3 to 6 years. Overall, 629 children participated in the present analysis

FFQs at the same follow-ups as 3-DFDs were also completed by parents or caregivers. The FFQ consisted of 158 food items divided into 12 food categories: milk and cheese, eggs, grains, vegetables, legumes, fruits, meats, fish, cakes, dressings and sauces, drinks, and snacks (Supplementary Table 1). Given the absence of an available European tool, this FFQ was developed by translating and adapting existing items to better reflect the dietary habits of the respective countries, rather than through an independent validation process. Average portion sizes were given in grams or milliliters for each food item. The parents were able to choose between different factors (0.5, 1, 1.5, 2) of these average portion sizes. In addition to this, the frequency of consumption was listed and the options to choose were: (never, 1, 2, or 3 times per day), (1, 2, 3–4, or 5–6 times per week), and (1 or 2–3 times per month). An example of a FFQ is depicted in Supplementary Fig. 1. The study population for the current analysis is visualized in Fig. 1.

### Mediterranean diet score

The KIDMED score consists of 16 items, with 12 items scoring 1 or 0 points for favorable food habits and 4 items scoring either 0 or -1 points for unfavorable ones. The total score ranges from -4 to 12 points, with scores ≤ 3 indicating poor adherence, scores 4–7 indicating medium adherence, and scores ≥ 8 indicating high adherence [18].

For this analysis, the KIDMED score was adapted to fit the CHOP FFQ and 3-DFD data. These adjustments were done according to the methodologies from Rei et al. who applied the original KIDMED on a 3-DFD [35], and Serra-Majem et al. who used dietary data from FFQ and 24h-recalls [17]. Questions about breakfast were modified to consumption independent of daytime, because the FFQ does not cover the timing of food intake. Furthermore, the question covering fast food restaurant visits was changed to consumption of red and/or processed meat, similar to the 14-item questionnaire for MD adherence used in other studies [36, 37]. The final scoring system is shown in Table 1. A logical disjunction was used to combine both scoring systems for FFQ and 3-DFD. This approach can be considered a type of “inclusive scoring” method, where the presence of a positive indicator in either of the sources is sufficient to produce a positive combined score. For example, if either the FFQ or 3-DFD would award one point for an item, the subsequent combined score would be 1. If both dietary protocols were to award a point, this would also correspond to a score of 1. If neither gives a point, the combined score would be 0. Inclusive scoring was used for the food items with negative scores as well.

This combined approach allows for a comprehensive assessment of MD adherence in children based on both FFQ and 3-DFD.

**Table 1:** Final combined score based on KIDMED – applicable for FFQ and 3-DFD

Item	Description	Score	Criteria for 1 point in FFQ	Criteria for 1 point in 3-DFD
1	Takes a fruit every day.	+ 1	≥ 1 servings per day	Listing of this item every day out of 3 days
2	Has a second fruit every day.	+ 1	≥ 2 servings per day	Second listing of this item every day out of 3 days
3	Has fresh or cooked vegetables once a day.	+ 1	≥ 1 servings per day	Listing of this item every day out of 3 days
4	Has fresh or cooked vegetables more than once a day.	+ 1	≥ 2 servings per day	Second listing of this item every day out of 3 days
5	Consumes fish regularly.	+ 1	at least 2-3x per week	at least 1 out of 3 days
6	Eats red and/or processed meat > 1 per week.	−1	> 1 per week	at least 1 out of 3 days
7	Eats pulses > 1 per week.	+ 1	> 1 per week	at least 1 out of 3 days
8	Eats pasta or rice almost every day.	+ 1	5 or more per week	Listing of this item every day out of 3 days
9	Has cereals or grains (bread, etc.) regularly.	+ 1	at least 2-3x per week	at least 1 out of 3 days
10	Eats nuts or seeds regularly.	+ 1	at least 2-3x per week	at least 1 out of 3 days
11	Consumes of olive oil regularly.	+ 1	at least 2-3x per week	at least 1 out of 3 days
12	Drinks, commercially produced drinks, soft drinks or juices > 1 per week.	−1	> 1 per week	at least 1 out of 3 days
13	Has an unsweetened dairy product (yoghurt, milk, cheese) daily.	+ 1	5 or more per week	Listing of this item every day out of 3 days
14	Has industrially baked goods or pastries, sweets, and candy daily.	−1	5 or more per week	Listing of this item every day out of 3 days
15	Has a second serving of unsweetened dairy product (yoghurt, milk, cheese) daily.	+ 1	≥ 2 servings per day	Second listing of this item every day out of 3 days
16	Has a second serving of industrially baked goods or pastries, sweets, and candy daily.	−1	≥ 2 servings per day	Second listing of this item every day out of 3 days

### Statistical methods

The mean KIDMED scores for the total study population and by country were calculated separately for FFQ and 3-DFD. Agreement in KIDMED scores between both diet assessment types were evaluated using concordance correlation using R package “DescTools” calculating Lin’s coefficient (ρ_c_). Additionally, Bland–Altman plots were created for each country and overall to visually assess the agreement between the two methods. Agreement intervals were calculated as the mean difference ± 1.96 standard deviations (SD), providing limits of agreement. Utilizing the above-described logical disjunction to take both scoring systems into account equally, we created a combined KIDMED score. The mean for the combined KIDMED score was calculated for the total study population, as well as separated by country and time point of follow-up. To evaluate the statistical significance of the differences between countries, multiple one-way Analysis of Variance (ANOVA) were conducted. To investigate which specific country differed from another following a significant ANOVA result, Tukey’s Honest Significant Difference (HSD) post-hoc test was applied for pairwise comparisons between levels. For comparison of the combined KIDMED score between follow-ups (longitudinal analysis for age), a linear mixed model with a random intercept for each child with time point as a fixed effect was conducted, followed by pairwise comparison of the consecutive follow-ups. In the case of binary variables, e.g., whether a child met (1) or did not meet (0) the criteria for receiving a point for one of the 16 items of the combined KIDMED score, a Pearson’s χ^2^ test or Fisher’s Exact Test was employed as applicable to compare (1) the five countries and (2) gender differences (independent groups). For comparison of these binary outcomes between the follow ups, a generalized estimating equation (GEE) was applied. An Intraclass Correlation Coefficient (ICC) was calculated to investigate how much of the variation in combined KIDMED scores is due to individual differences across years in each country. For this purpose, a linear mixed-effects model was done for each country, including a fixed intercept to estimate the overall average KIDMED score and a random intercept for each child to account for individual differences from that average. This allowed us to estimate both between-child and within-child variance and subsequently calculate the ICC.

Data preparation as well as statistical analyses were done using R Studio Version 2024.04.2 + 764. Statistical significance was assumed at a maximum error probability of 0.05.

### Ethics

The trial was conducted in accordance with the Declaration of Helsinki, approved by the ethics committee from each participating site. Parents or legal guardians signed informed consent to participate in the study.

## Results

### Study population

The characteristics of the study population, consisting of all subjects who attended at least one time point between the ages of 3 and 6 years with both FFQ and 3-DFD assessment data, are presented in Table 2. In total, 629 children participated, with 1,640 dietary data assessments over all time points. The largest proportions of children were from Italy (29.4%) and Spain (26.7%). The average paternal BMI (26.0 ± 3.6) was higher than the maternal pre-pregnancy BMI (23.4 ± 4.1). At age 3 years, 6 children were classified to have obesity based on WHO standards of > + 2 SD. Most parents (88.7%) showed medium to high educational levels according to re-categorized ISCED [38].

**Table 2:** Characteristics of the study population with at least one combined food frequency questionnaire and 3-Day food diary assessment between 3 and 6 years of age

Characteristic	Number of participants *n* = 629
*Country (n(%))*	
Belgium	67 (10.7%)
Germany	92 (14.6%)
Italy	185 (29.4%)
Poland	117 (18.6%)
Spain	168 (26.7%)
*Gender (n (%))*	
Female	332 (52.8%)
Male	297 (47.2%)
*Children’s BMI*^*1*^ *(Mean ± SD)*	
at 3 years *n* = 578	15.98 ± 1.33
at 4 years *n* = 556	15.88 ± 1.36
at 5 years *n* = 540	15.86 ± 1.61
at 6 years *n* = 552	15.98 ± 1.97
Overweight/Obesity^2^ at 3 years, n (%)	25 (4.3%)
*Parental BMI*^*1*^ *(Mean ± SD)*	
Mother (pre-pregnancy) *n* = 606	23.41 ± 4.13
Father (1 year after birth) *n* = 537	26.01 ± 3.62
*Parental highest education achievement (ISCED* ^3^ *) (n(%))*	
No/low	71 (11.3%)
middle	314 (50.0%)
High	243 (38.7%)

^1^Children’s & parental BMI for all participants, independent of available dietary data at that follow-up

^2^Overweight/Obesity according to WHO standards of > + 1 SD; SD = standard deviation

^3^ISCED re-categorised: 1–2 = no/low; 3–4 = middle; 5–6 = high [38].

### Comparison of dietary assessment tools

Table 3 presents means and standard deviations (SD) of KIDMED scores for FFQ and 3-DFD, as well as their concordance correlation coefficient (ρ_c_ with 95% confidence interval) for each country over the ages studied. In addition, the combined KIDMED scores and the categorization of MD adherence are shown. The KIDMED score based on FFQ was considerably higher than the 3-DFD-based score (Δ = 1.53), while the concordance between KIDMED score from FFQ and 3-DFD was moderate to poor (ρ_c_ all countries = 0.43 [95% CI 0.40, 0.47]). Within countries the lowest concordance was seen for Spain ρ_c_ = 0.15 [0.10, 0.21]. In Supplementary Fig. 2, Bland-Altman plots with the correlation between FFQ and 3-DFD KIDMED scores overall and stratified by country are visualized.

**Table 3:** Average KIDMED scores based on dietary assessment tool (FFQ, 3-DFD or combined) in means with SD, the concordance correlation coefficient ρc for comparison of FFQ and 3-DFD, as well as the categorization of adherence per country based on the combined KIDMED score

Country	FFQ-based KIDMED score	3-DFD-based KIDMED score		ρ_c_ 95% CI	Combined KIDMED score		Categorization of adherence to the MD^1^ *n* (%)
Belgium	1.39 ± 2.03	0.30 ± 1.81		0.33 [0.20, 0.46]	1.96 ± 2.07		Poor: 106 (78%) Medium: 30 (22%) High: 0 (0%)
Germany	2.69 ± 2.05	0.72 ± 2.03		0.26 [0.17, 0.34]	3.13 ± 2.01		Poor: 120 (56%) Medium: 91 (43%) High: 3 (1%)
Italy	4.25 ± 1.80	3.50 ± 1.90		0.25 [0.18, 0.31]	5.00 ± 1.60		Poor: 102 (17%) Medium: 480 (79%) High: 24 (4%)
Poland	1.39 ± 2.08	− 0.67 ± 1.60		0.19 [0.12, 0.25]	1.65 ± 2.12		Poor: 253 (81%) Medium: 60 (19%) High: 0 (0%)
Spain	4.47 ± 1.92	2.17 ± 1.85		0.15 [0.10, 0.21]	4.54 ± 1.70		Poor: 103 (28%) Medium: 257 (69%) High: 11 (3%)
All countries	3.31 ± 2.33	1.78 ± 2.45		0.43 [0.40, 0.47]	3.76 ± 2.28		Poor: 684 (56%) Medium: 918 (42%) High: 38 (2%)

^1^Based on cut-off values (≤ 3 poor, 4–7 medium, ≥ 8 high adherence) applied on the combined KIDMED score.

*FFQ* food frequency questionnaire, *3-DFD*  3 day food diary, *KIDMED* childhood mediterranean diet score, ρ_c_ concordance correlation coefficient,* CI*  confidence interval, *MD* mediterranean diet

### Comparison of the five European countries per assessment tool

The mean FFQ-based KIDMED scores were significantly different (all *p* < 0.01) between all countries except the two non-Mediterranean countries Belgium and Poland (Δ = 0,00 *p* = 1.000), and between the Mediterranean countries Spain and Italy (Δ = 0.22, *p* = 0.425) (Supplementary Table 2). The greatest differences were found between Spain and Belgium (Δ = 3.08), and Spain and Poland (Δ = 3.08). Similarly, the 3-DFD-based KIDMED scores was significantly different between countries (*p* < 0.01), with the exception between the two non-Mediterranean countries Germany and Belgium (Δ = 0.42, *p* = 0.237). The greatest differences occurred between Poland and Italy (Δ = − 4.17) and Italy and Belgium (Δ = 3.20). For the combined KIDMED score, significant differences (all *p* < 0.01) were observed between almost all countries, except for the comparison of the two non-Mediterranean countries Poland and Belgium (Δ = − 0.31, *p* = 0.485).

### Contribution of dietary assessment tools to the combined KIDMED score

**Fig. 2:**
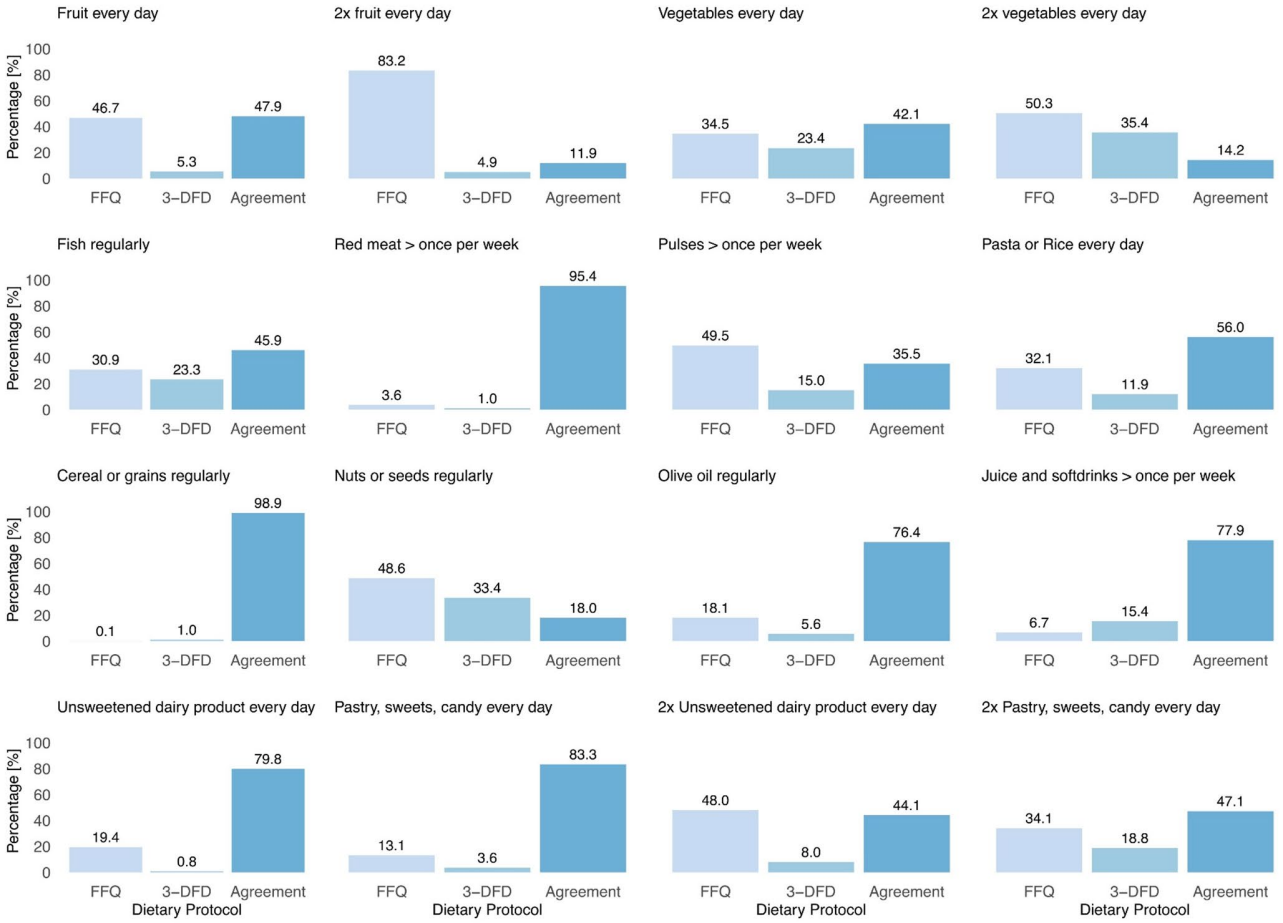
Proportion of all participants reaching the item threshold for the combined KIDMED score via exclusively FFQ (1|0), 3-DFD (0|1), or in agreement (1|1). *FFQ* food frequency questionnaire, *3-DFD * 3 day food diary

As large differences were found between the FFQ- and 3-DFD-based KIDMED score, we also analysed the 16 included single items. Figure 2 shows the percentage contribution to the combined KIDMED score by item and by dietary assessment method, indicating whether the FFQ (1|0), the 3-DFD (0|1), or both in agreement (1|1) assigned the combined KIDMED score point. This figure highlights discrepancies in the scoring of specific food items between FFQ and 3-DFD. For instance, almost 95% of the children were identified by FFQ to eat *fruit every day* while 53% were consuming fruits daily in the 3-DFD. There is an agreement (1|1) in almost 48% of the daily fruit consumption, i.e., the participant received a point in FFQ and 3-DFD respectively. Conversely, intake of *red and processed meat*, *cereal or grains*, *juice and soft drinks*, *unsweetened dairy products every day*, and *pastry*,* sweets and candy every day*, are identified similarly by both protocols. Lastly, relevant *fish* intake was identified in over 69% of all observations by the 3-DFD compared to almost 77% by FFQ.

### Combined KIDMED score: tracking over time

**Fig. 3:**
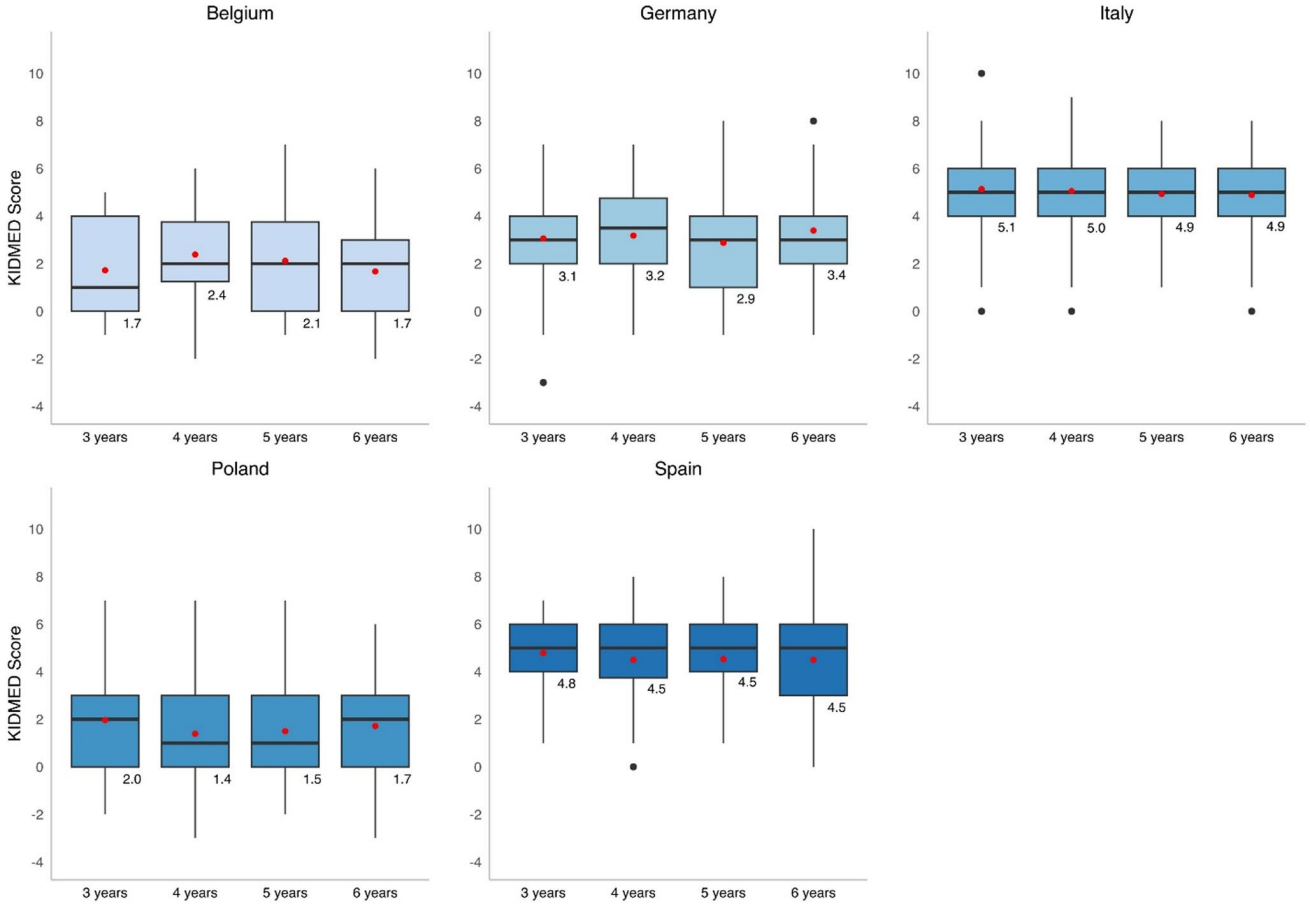
Combined KIDMED score per country for each follow-up. Red dot represents the mean KIDMED score, while the black horizontal line represents the median

Examination of the combined KIDMED score over the follow-ups revealed that the scoring remained stable across all countries over the four time points (Fig. 3). No significant differences were found between the combined KIDMED scores across different time points within each country, except for Poland with a decrease between the ages of 3 and 4 years. However, ICC values indicate varying levels of individual score stability across the five countries with Germany: 0.620, Belgium: 0.604, Italy: 0.429, Poland: 0.564, and Spain: 0.493.

### Single items of the combined KIDMED score

A significant increase of 10.2% in the number of children consuming *fish regularly* (as defined in Table 1) was observed between the ages of 3 and 5 years (*p* = 0.015) and an increase of 12.2% between the ages of 3 and 6 years (*p* = 0.002). At the 5-year follow-up, 7% more children were consuming a second serving of sweets compared to the 3-year baseline (*p* = 0.037). All other items showed no significant differences between time points. The results are presented in Supplementary Fig. 3.

There was no significant difference between boys and girls in the consumption of each food item except for dairy (*2x unsweetened dairy product every day*). In this case, 6% more boys than girls consumed an amount reaching the threshold for this item (*p* = 0.01) (Supplementary Fig. 4). In total, there was no significant gender difference in the combined KIDMED score.

**Fig. 4:**
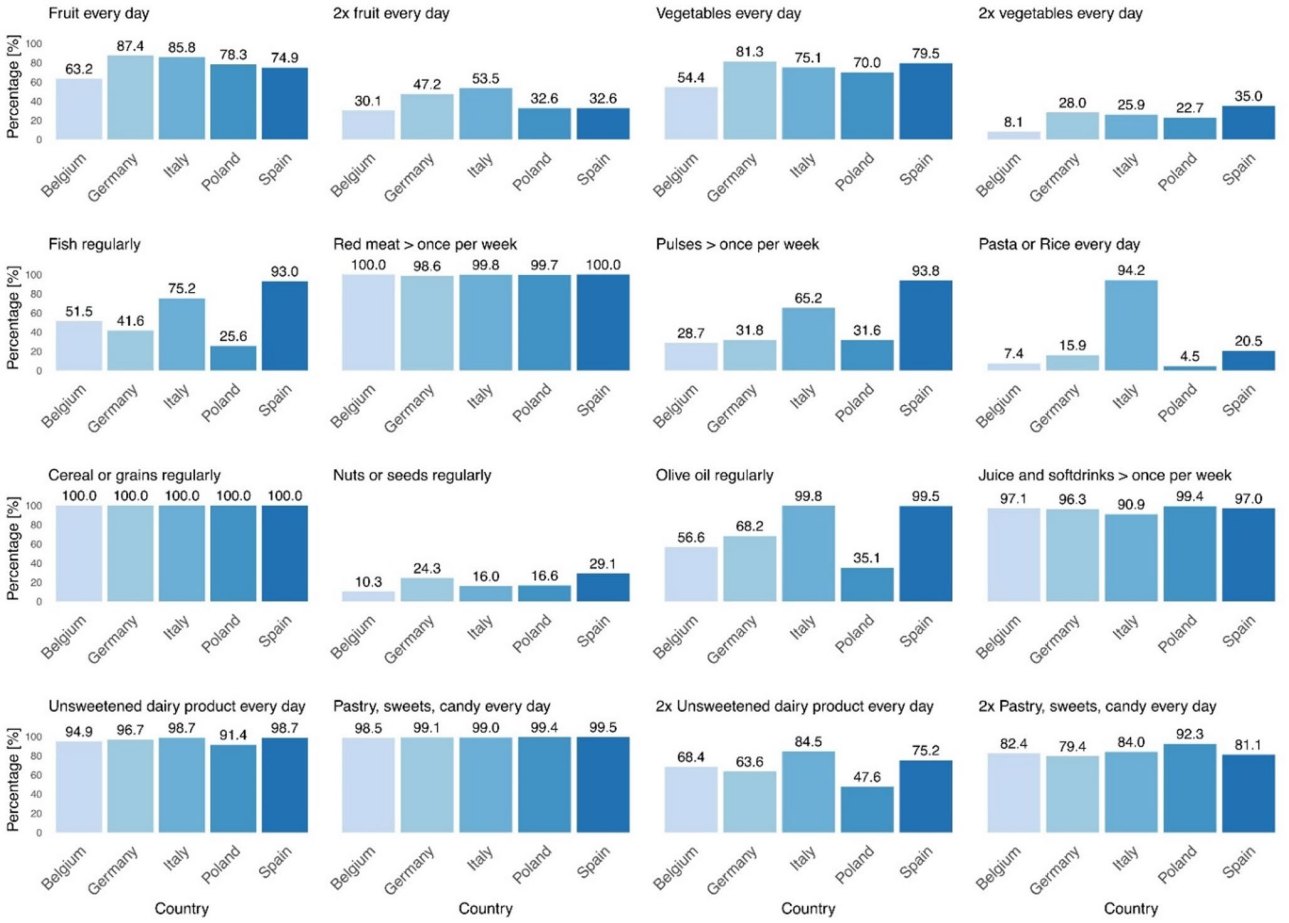
Proportion (%) of children reaching the threshold of a KIDMED score item in each country

The percentages of children meeting the scoring thresholds for each item by country over the entire time period and the differences between countries are shown in Fig. 4 and Supplementary Table 3. In the assessment of *daily fruit* consumption, Germany had the highest percentage of children meeting the threshold at 87%, while Belgium showed the lowest proportion of 63% (*p* < 0.0001). Only 30% of Belgian children had a *second serving of fruit* daily, compared to 54% of Italian children (*p* < 0.0001). Regarding *daily vegetable* consumption, Germany and Spain had the highest proportions of children meeting this threshold, at 81% and 80% respectively, with Belgium trailing at 54% (both *p* < 0.0001). For the *second serving of vegetables* daily, only 8% of Belgian children met the threshold, compared to 35% of Spanish children (*p* < 0.0001). There were significant differences in *fish* consumption among countries: 93% of Spanish children consumed *fish* regularly, whereas only 26% of Polish children did (*p* < 0.0001). Regular consumption of *nuts and seeds* was seen in 29% of Spanish children, but only in 10% of Belgian children (*p* < 0.0001). Nearly all Italian and Spanish children regularly consumed *olive oil*, while this was true for only 35% of Polish children (*p* < 0.0001). The majority of Italian children consumed *pasta and rice* almost every day while all other countries showed a significantly lower percentage of children achieving this threshold (*p* < 0.0001). In all five countries, juices are consumed by nearly all children, with minor but noteworthy variations between Poland (99%) and Italy (91%) (*p* < 0.0001), as well as Spain (97%) and Italy (*p* = 0.004). For a *second serving of unsweetened dairy products* daily, 85% of Italian children and 75% of Spanish children met the threshold compared to 48% of Polish children (*p* < 0.0001). Additionally, a *second serving of pastries*,* sweets*,* or candies* daily is consumed by 92% of Polish children and 79% of German children (*p* < 0.0001). No notable differences were observed among the countries for daily consumption of *red meat* and *cereals and grains*, as most children consume these food items regularly.

## Discussion

The analysis presented here indicates that the KIDMED score is markedly affected by the dietary assessment tool used, i.e., FFQ and 3-DFD. Since both methodologies offer unique strengths and areas of focus in dietary analysis, this variability does not imply that one is less reliable than the other in assessing dietary patterns. Hence, we created a combined KIDMED score to take advantage of both assessment methods and evaluated this approach in the CHOP trial. The subsequent analyses demonstrated that each method captures distinct dietary behaviors – as evidenced by the analysis of the individual items across follow-ups, and stratified by country or gender – and contributes uniquely to the combined KIDMED score. Therefore, creating a score that is based on both FFQ and 3-DFD advances the value of dietary assessment methodologies.

### Comparison of the FFQ, 3-DFD, and combined KIDMED score

We observed that 3-DFD consistently yielded lower KIDMED scores than basing it on FFQ – there by suggesting a lower adherence to the MD – across all time points and countries examined. These differences are also quantified in the Lin´s concordance correlation coefficient, which showed a poor to moderate correlation between FFQ and 3-DFD [39]. Discrepancies may be attributed to the inherent differences in data collection tools. FFQs may overestimate dietary adherence due to reliance on memory and also a tendency of respondents to report socially desirable behaviors, which in this case could lead to a higher KIDMED score [40, 41]. A more immediate and detailed record of consumption is provided by 3-DFD, however, potential under- or overreporting and the “observer effect” might lead to bias in reporting the actual dietary pattern as well [40, 41]. Previous studies have compared FFQ results with other dietary assessment tools, e.g., to calculate the Alternative Healthy Eating Index, where FFQ also generated higher scores compared to 24h-recalls [42, 43]. Kowalkowska et al. compared FFQ data with three-day unweighted food records and found differences in the nutritional value of diets, with higher estimations of energy and nutrient intake with the FFQ [44]. FFQ and 3-DFD have inherent strengths and unavoidable weaknesses. However, combining them could enhance their advantages while reducing their disadvantages and thereby result in a more representative dietary evaluation.

The KIDMED score comprises 12 positively scored items and 4 negatively scored items. Due to a generally higher estimation with the FFQ, the positively scored items disproportionately influence the overall KIDMED score compared to the negatively scored items. This is reflected in the combined KIDMED score, as the inclusive scoring method emphasizes the predominantly positive items. Hence, the combined KIDMED score exhibits more high scores and a higher mean compared to the FFQ and 3-DFD. In contrast, it is also more likely to detect the negative points in the inclusive scoring method, such as frequent consumption of red meat or sweets and pastries, which usually may not be as easily detected in the 3-DFD. This is due to the three-day recording period, as it could provide a limited representation of habitual dietary intake, as participants may have day-to-day fluctuations in certain food intakes. Consequently, it may underrepresent or overrepresent specific foods based on their consumption during the recorded period. This limitation is well documented in dietary assessment methodology [45]. Despite this, a recent analysis showed that 3–4 days of dietary records are considered to be sufficient for a reliable estimate of general macronutrient, as well as meat and vegetable intake [46]. One further issue that might remain unresolved is a possible overreporting of fruits and vegetables in FFQs [47]. Contrary to expectations, our investigation into the contributions of dietary assessment tools to the combined KIDMED score revealed discrepancies with established knowledge. We anticipated that the 3-DFD would be less effective in capturing the intake of non-daily consumed food such as fish, nuts, or seeds [41]. However, our results indicate that 23% of *fish* and 33% of *nuts or seeds* consumption were reported exclusively in 3-DFD while not being captured by FFQ. A potential limitation of the FFQ data is the lack of validation. However, FFQ validation studies typically focus on assessing portion sizes. Because our analysis uses only frequency data and not portion sizes, this limitation is unlikely to have substantial influence on the results.

### Higher adherence in mediterranean countries

Our findings demonstrate that participants from Mediterranean countries (Italy and Spain) consistently have higher FFQ based and combined KIDMED scores. In line with our findings, a systematic review from 2016 found that children living in the Mediterranean regions tend to consume more fruit and vegetables than children in non-Mediterranean countries. However, the overall adherence to the MD remains low due to the high consumption of energy-dense and sodium-rich foods [10]. Similarly, our study revealed that while adherence to the MD in Mediterranean countries was higher, the mean KIDMED score per country never reached the cut-off value of ≥ 8 for “high adherence”, indicating potential for dietary improvements.

Children in Belgium displayed a higher KIDMED score in two out of the three scores compared to Poland. These results are consistent with a publication by Sezaki et al. using data from 2009 to study the effect of a MD on healthy life expectancy [48, 49]. The findings from that study also indicated that children in Belgium had a higher MD adherence than those in Germany and Poland. However, our results differ, showing higher scores in Germany than in Belgium and Poland. This discrepancy might be influenced by inclusion of the entire population by Sezaki et al., whereas our population consisted of children aged 3–6 years only [48].

### Stability of dietary patterns over time

A systematic review revealed that dietary patterns of children and young adults trend towards a “Westernized” diet while the adherence to a MD pattern is decreasing [50]. In the present study, the individual and overall dietary pattern and adherence to the MD was relatively stable over time. ICC values ranged from 0.429 to 0.620, indicating poor to moderate reliability for individual diet scores across time points [51]. The mean scores observed across follow-ups further support the overall consistency of diet scores, with results showing no significant variation over time within each country, which is in agreement with previous publications reporting tracking of dietary patterns acquired early in life [52–54].

### Single food items of the combined KIDMED score: longitudinal analysis

In this analysis, the number of children reaching the threshold for the respective scoring item was stable for most food items over time, indicating that dietary patterns established at a young age tend to persist when they grow older [55] and have the potential to remain relatively stable throughout the life course [56].

The number of children with regular fish consumption is low but increases across follow-ups. This trend might be attributed to dietary recommendations for pregnant women and children suggesting that fish and seafood should be consumed regularly. Concerns about potential adverse effects of heavy metals and biogenic amines found in fish [57] might lead families to offer fish less frequently to young children. However, we found a high consumption of negatively scored food items such as red meat, pastries/candies, or soft drinks with no change over the follow-ups. Almost all children in the CHOP study consume red meat more than once a week even though high red meat consumption has been linked to adverse health effects [58, 59]. Similarly, sugary beverages are consumed by almost all children on a regular basis, even though they contribute to the risk of obesity, diabetes, and other risks [60]. In addition, almost all children ate candies at least once every day – approximately a quarter consumed it twice per day, in spite of documented adverse health effects [61]. Given that the contribution of ultra-processed foods to adults’ caloric intake has approximately doubled since the early 2000s, it is plausible that children’s intake of these foods may have followed a similar upward trajectory [62]. Furthermore, a recent publication reported a decline in children’s adherence to the Mediterranean diet over the past two decades, suggesting that our data may not fully reflect current dietary patterns and that contemporary KIDMED scores could be lower [63].

### Single food items of the combined KIDMED score: country differences

The data indicated that children in the Mediterranean countries Italy and Spain, in addition with Germany had the highest fruit and vegetable intake. A high regular consumption of fish in Spain and Italy contrasted with the lower intakes in Germany and Poland. On the other hand, red meat consumption more than once per week was consistently high across all surveyed countries. The regular intake of pulses was notably higher in Spain and relatively low in other countries. Italy’s high daily consumption of *pasta or rice* reflected the country’s traditional cuisine [64, 65]. Regular olive oil consumption was exceptionally high in Italy and Spain, which is consistent with the MD pattern. All countries reported high regular consumption of cereals or grains. However, the intake of nuts or seeds was uniformly low, even though regular consumption of nuts and seeds has been associated with health benefits [66]. The daily consumption of unsweetened dairy products was high across all countries, suggesting a high adherence to dietary recommendations for dairy intake [67]. A frequent intake of juice and soft drinks more than once per week was prevalent across all five countries.

### Strengths and limitations

The present analysis utilizes longitudinal data from four time points across five European countries, where parents completed two dietary assessment methodologies for their children: the FFQ and the 3-DFD. A strength of this analysis is the unique opportunity to compare and combine these dietary assessment tools and to calculate a dietary score of MD adherence. This approach enables a more detailed analysis and evaluation of the dietary composition of young preschool children from five European countries, three of which are non-Mediterranean, over a 3-year period by examining the consumption of 16 food items that are crucial components of the MD. However, there are also limitations to this analysis. The data used is from 2006 to 2009, and dietary habits of today’s 3- to 6-year-old children may have changed. In addition, the applied KIDMED scoring system is based on frequencies and not on actual amounts of consumed food items which may lead to a distortion of the real adherence to the MD. Parents’ dietary habits, timing of solid foods introduction, and other factors may influence children´s dietary pattern. These were not part of this analysis, as the main focus of this publication was combining FFQ and 3-DFD data, the influence of these assessment tools on the combined KIDMED score, the evaluation of country differences, and the description of the combined KIDMED score stability over time.

## Conclusion

The present analysis provides valuable insights into the effect of dietary assessment methodology on the KIDMED scoring. It further adds knowledge about the MD adherence of children from five different European countries. The combination of FFQ and 3-DFD resulted in a higher KIDMED score with a possible tendency to overestimate healthy food intake. Mediterranean countries showed higher adherence to the MD than non-Mediterranean countries. Furthermore, MD adherence in early childhood remained poor to moderate and mean combined KIDMED scores stable over time, suggesting that dietary patterns established at a young age are likely to persist. We conclude that this approach to combine both FFQ and 3-DFD to a combined KIDMED score is able to capture more details – positive and negative – of different consumed food items.

## Supplementary Information

Below is the link to the electronic supplementary material-

Supplementary Material 1

## Data Availability

According to the informed consent, original data sharing with other researchers requires a written data sharing agreement. Written requests to access the data with a reasonable interest may be submitted to: office.koletzko@med.uni-muenchen.de.

## Abbreviations

3-DFD: 3-day food diary
ANOVA: Analysis of variance
BMI: Body mass index
CHOP: European childhood obesity project
FFQ: Food frequency questionnaire
ICC: Intraclass correlation coefficient
KIDMED: Mediterranean diet quality index for children and adolescents
MD: Mediterranean diet
