# Supplementary material for: Mediterranean dietary pattern analysis combining two nutritional assessment tools in children aged 3 to 6 years in five European countries
Source: Eur J Nutr. 2026 Apr 15;65(3):118. doi: 10.1007/s00394-026-03930-y (PMC13083398; doi:10.1007/s00394-026-03930-y)
Supplement: Supplementary file 1 — Supplementary Material 1 [file 394_2026_3930_MOESM1_ESM.docx]

**Supplementary Material**

Supplementary Table 1: Food Groups and their included foods from Food Frequency Data and 3-Day Food Diary, for KIDMED categorization.

| Food Group | Included Foods from FFQ | Included Foods from 3-DFD |
| --- | --- | --- |
| Fruit Every Day | Apple, Pear, Banana, Orange, Tangerine, Peaches, Apricots, Strawberry, Plumes, Cherries, Blackberries, Pineapple, Kiwi, Grapes, Avocado, Melon, Watermelon, Medlars, Grapefruits, Persimmon, Dried figs, Dried plum | berries & wild, citrus fruits, malaceous & stone fruits, tropical fruits |
| Vegetables Every Day | Cauliflower, Artichokes, Asparagus, Broccoli, Swiss chard, Red beetroots, Yellow pumpkin, Lettuce, Courgettes, Cucumbers, Fennel, Cultivated mushrooms, Tomatoes for salad, Raw spinach, Mixed vegetables deep frozen (peas, carrots, beans, corn), Carrots, Onions, Yellow peppers, Green olives | Cruciferous vegetables, fruiting vegetables, leafy green vegetables, sprout, roots, salad vegetables, soups & vegetable dishes |
| Fish Regularly | Cod or Hake, Sole, Clams, Mussels, Prawn, Octopus, Cuttlefish, Salmon, Fresh herrings, Smoked mackerel, Tuna, Gilthead, Fresh anchovy, Swordfish, Tuna in olive oil | Lean fish and seafood |
| Red and Processed Meat | Raw lamb, Rabbit, Veal (raw fillet, shank, minced), Horse, Entrecote beef, Thin pork, Semi-fat pork (pork côtelette), Pork chop, Fat pork, Pork sausage, Tinned corned beef, Boiled jellied cattle, Dry-salted beef, Pork sausages, Bologna, Mortadella, Salami, Ham, Speck, Visceral fresh meat, Beef goulash, Spiced sausage (salsichón), Chistorra, Boiled sausages with meat (10–20% fat), Sausage meat dumpling | Processed meat, red meat |
| Pulses More Than Once a Week | Raw dried beans, Tinned drained beans (Borlotti style), Green beans (raw), Fresh raw peas | Leguminous vegetables |
| Pasta or Rice Every Day | Raw semolina pasta, Rice | Pasta, rice dishes:  Whole-grain and refined grain |
| Cereals and Grains | White bread, White roll, Infantile cereals, Cornflakes, Muesli, Crackers, Crisp bread, Unleavened bread, Wholemeal bread | Processed cereal products, refined grains, whole grains |
| Nuts Regularly | Pine seeds, Pistachios, Walnuts | Nuts and seeds |
| Olive Oil | Olive oil | Olive oil |
| Soft Drinks and Juice | Fruit juice, Cola and lemonades, Fruit syrup to be added to water | Soft drinks, flavored milk, fruit juices |
| Dairy | Cow's pasteurized whole milk, Cow's pasteurized partially skimmed milk, Growing milk, Skimmed milk, Whole yoghurt, Skimmed plain yoghurt, Skimmed fresh cheese, Fresh cheese, Mozzarella from cow, Melted cheese, Parmesan, Emmenthal, Stracchino (very soft and fresh cheese), Brie cheese, Spain cheese | Milk and milk products not sweetened – cream, cream cheese, curd, hard cheese, milk full fat/semi skimmed & skimmed, semihard cheese, soft cheese, yoghurt |
| Baked Goods and Candy | Biscuits, Wholemeal biscuits with fruit, Soya biscuits, Croissant, Candies, Pure chocolate, Milk chocolate, Coconut bar covered with chocolate, Manufactured ice cream with vanilla and chocolate, Vanilla or fruit ice cream, Ice lolly, Jam, Honey, Sugar (saccharose), Tart with fresh fruit, Brioche, Butter cake, Tartlet, Assorted pastries, Profiteroles, Vanilla cake, Tiramisù, Vanilla or chocolate pudding, Chocolate biscuits, Chewing gum, Waffles, Thick chocolate, Ovomaltina | Cakes, pastries, biscuits, infant cereals sweetened, milk products sweetened, salty snacks, sugar/honey, sweet main dish, sweets, instant cereals sweetened |


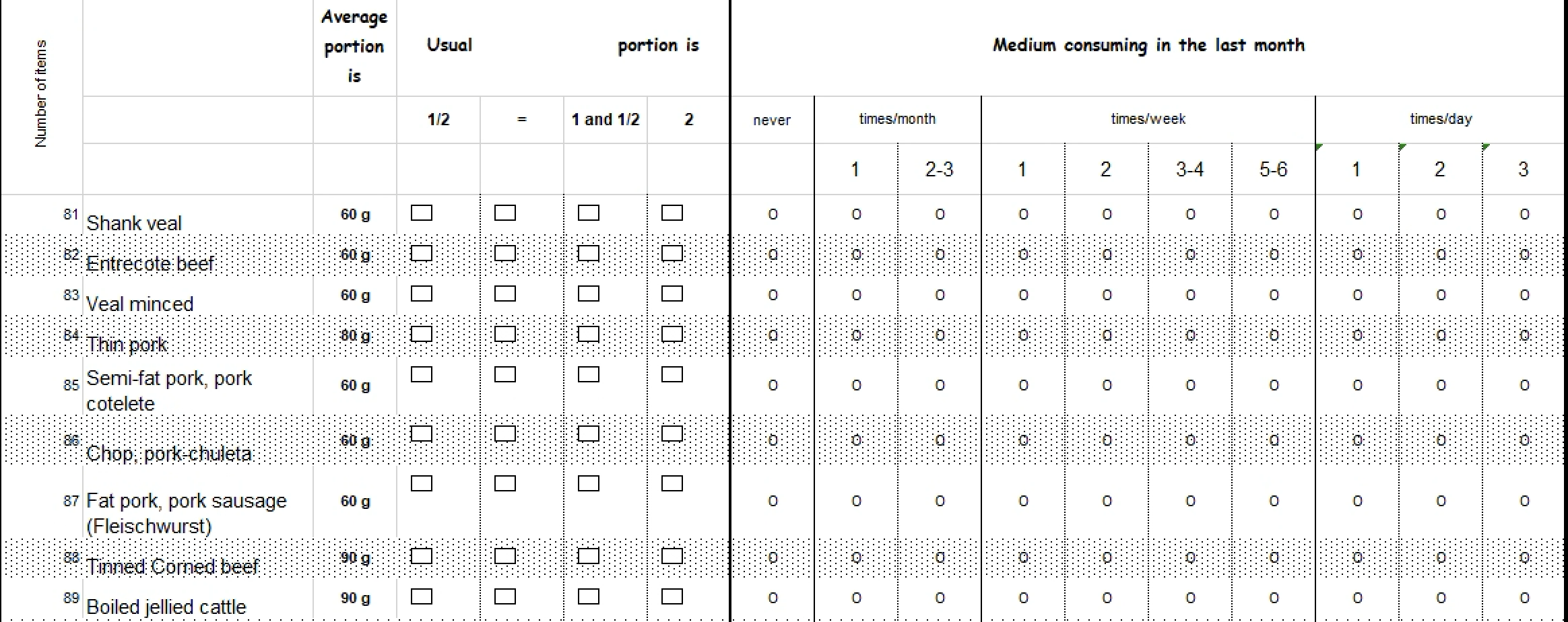


Supplementary Figure 1 Example of the Food Frequency Questionnaire (FFQ)

**
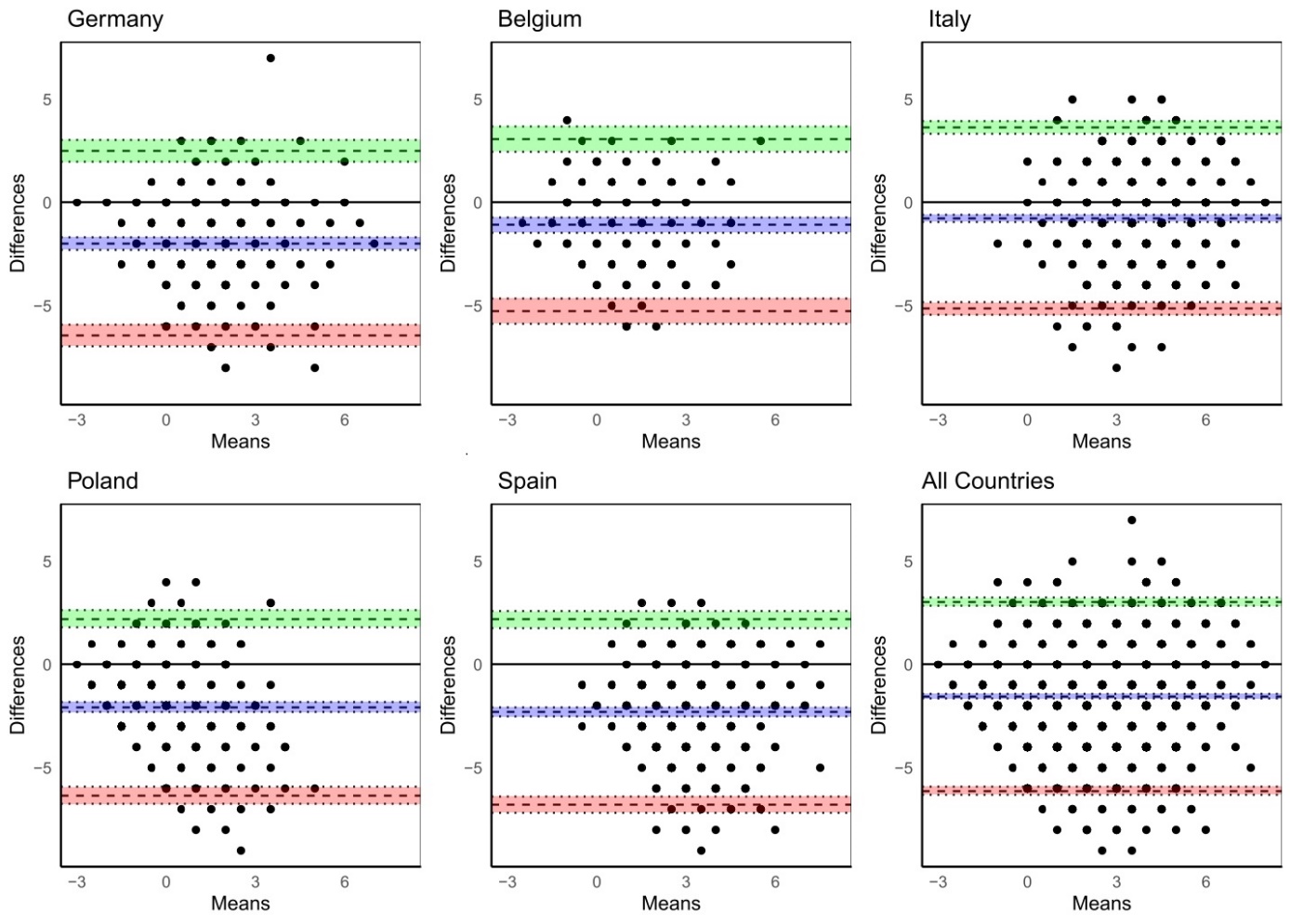
**

Supplementary Figure 2 Bland-Altman plots showing the correlation between Food Frequency Questionnaire and 3-Day Food Diary KIDMED scores for each country and overall.

Supplementary Table 2: Scoring differences (Δ) based on ANOVA and Tukey´s HSD between countries for combined KIDMED score, Food Frequency Questionnaire (FFQ) score and 3-Day Food Diary (3-DFD) score, respectively.

|  | **Combined KIDMED Score** | | **FFQ KIDMED Score** | | **3-DFD KIDMED Score** | | |
| --- | --- | --- | --- | --- | --- | --- | --- |
|  | **Δ** | **p-value** | **Δ** | **p-value** | **Δ** | | **p-value** |
| Germany-Belgium | 1.17** | <0.01 | 1.30** | <0.01 | 0.42 | 0.237 | |
| Italy-Belgium | 3.05** | <0.01 | 2.86** | <0.01 | 3.20** | <0.01 | |
| Poland-Belgium | -0.31 | 0.485 | 0.00 | 1.00 | -0.97** | <0.01 | |
| Spain-Belgium | 2.59** | <0.01 | 3.08** | <0.01 | 1.87** | <0.01 | |
| Italy-Germany | 1.88** | <0.01 | 1.56** | <0.01 | 2.78** | <0.01 | |
| Poland-Germany | -1.47** | <0.01 | -1.30** | <0.01 | -1.39** | <0.01 | |
| Spain-Germany | 1.42** | <0.01 | 1.78** | <0.01 | 1.45** | <0.01 | |
| Poland-Italy | -3.35** | <0.01 | -2.86** | <0.01 | -4.17** | <0.01 | |
| Spain-Italy | -0.46** | <0.01 | 0.22 | 0.425 | -1.33** | <0.01 | |
| Spain-Poland | 2.89** | <0.01 | 3.08** | <0.01 | 2.84** | <0.01 | |
| ** p-value < 0.05; ** p-value < 0.01; *** p-value < 0.001* | | | | | |  | |


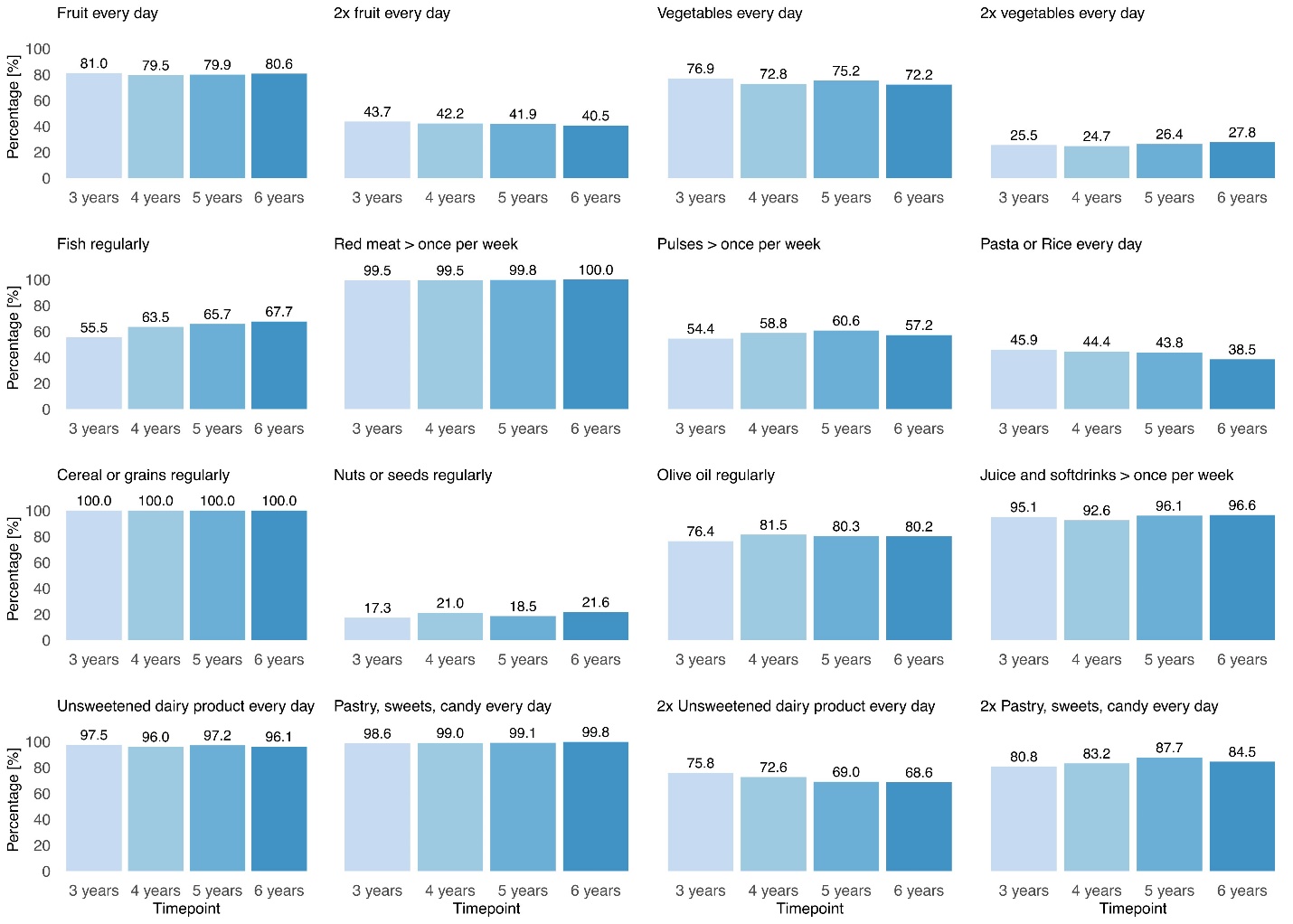


Supplementary Figure 3 Proportion of participants meeting the criteria of a KIDMED score item over time.


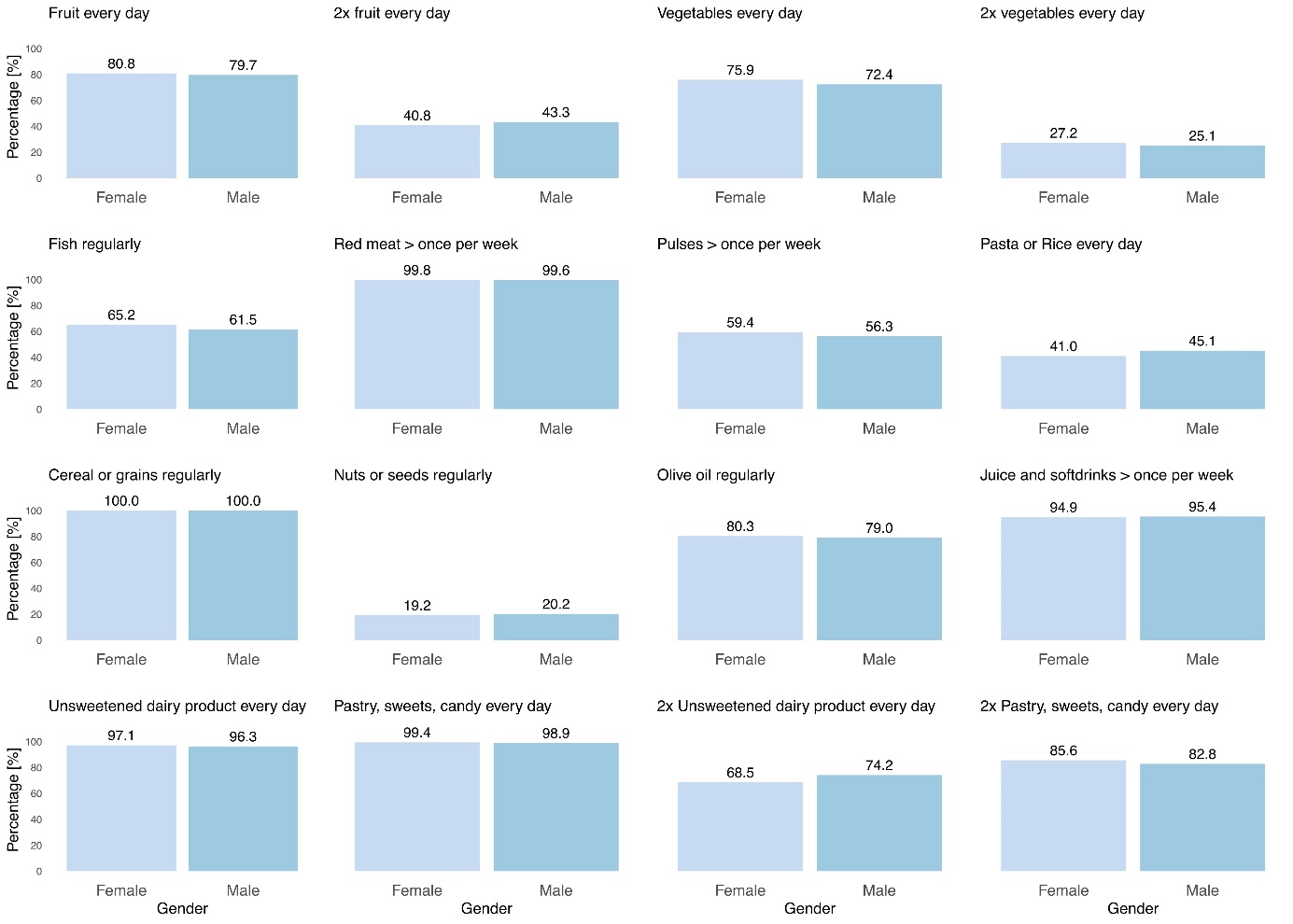


Supplementary Figure 4 Gender differences in meeting the criteria of a KIDMED score item.

Supplementary Table 3: Pearson's χ2 test/Fisher exact test results of the country comparison for reaching the threshold of each combined score item.

| **Comparison** | **Combined** | **diff** | **p adj*** | **Combined** | **diff** | **p adj*** | **Combined** | **diff** | **p adj*** | **Combined** | **diff** | **p adj*** |
| --- | --- | --- | --- | --- | --- | --- | --- | --- | --- | --- | --- | --- |
| Germany-Belgium | Fruit every day * | 0.241 | <0.0001 | 2x fruit every day * | 0.170 | 0.025 | Vegetables every day * | 0.269 | <0.0001 | 2x vegetables every day * | 0.199 | <0.0001 |
| Italy-Belgium |  | 0.226 | <0.0001 |  | 0.233 | <0.0001 |  | 0.207 | <0.0001 |  | 0.178 | <0.0001 |
| Poland-Belgium |  | 0.150 | 0.013 |  | 0.024 | 1.000 |  | 0.156 | 0.002 |  | 0.146 | 0.004 |
| Spain-Belgium |  | 0.117 | 0.131 |  | 0.025 | 1.000 |  | 0.251 | <0.0001 |  | 0.270 | <0.0001 |
| Italy-Germany |  | -0.016 | 1.000 |  | 0.063 | 1.000 |  | -0.062 | 0.787 |  | -0.021 | 1.000 |
| Poland-Germany |  | -0.091 | 0.106 |  | -0.146 | 0.010 |  | -0.113 | 0.046 |  | -0.054 | 1.000 |
| Spain-Germany |  | -0.125 | 0.005 |  | -0.146 | 0.006 |  | -0.018 | 1.000 |  | 0.070 | 0.988 |
| Poland-Italy |  | -0.075 | 0.050 |  | -0.209 | <0.0001 |  | -0.051 | 1.000 |  | -0.032 | 1.000 |
| Spain-Italy |  | -0.109 | <0.0001 |  | -0.209 | <0.0001 |  | 0.044 | 1.000 |  | 0.091 | 0.030 |
| Spain-Poland |  | -0.033 | 1.000 |  | 0.000 | 1.000 |  | 0.095 | 0.053 |  | 0.124 | 0.006 |
| Germany-Belgium | Fish regularly * | -0.099 | 0.891 | Red meat > once per week ^+^ | 0.014 | 1.000 | Pulses > once per week * | 0.031 | 1.000 | Pasta or rice every day * | 0.085 | 0.291 |
| Italy-Belgium |  | 0.238 | <0.0001 |  | 0.002 | 1.000 |  | 0.365 | <0.0001 |  | 0.869 | <0.0001 |
| Poland-Belgium |  | -0.259 | <0.0001 |  | 0.003 | 1.000 |  | 0.030 | 1.000 |  | -0.029 | 1.000 |
| Spain-Belgium |  | 0.415 | <0.0001 |  | 0.000 | - |  | 0.651 | <0.0001 |  | 0.131 | 0.008 |
| Italy-Germany |  | 0.337 | <0.0001 |  | -0.012 | 0.870 |  | 0.334 | <0.0001 |  | 0.783 | <0.0001 |
| Poland-Germany |  | -0.160 | 0.002 |  | -0.011 | 1.000 |  | -0.001 | 1.000 |  | -0.114 | <0.0001 |
| Spain-Germany |  | 0.514 | <0.0001 |  | -0.014 | 0.830 |  | 0.620 | <0.0001 |  | 0.046 | 1.000 |
| Poland-Italy |  | -0.497 | <0.0001 |  | 0.002 | 1.000 |  | -0.336 | <0.0001 |  | -0.898 | <0.0001 |
| Spain-Italy |  | 0.177 | <0.0001 |  | -0.002 | 1.000 |  | 0.286 | <0.0001 |  | -0.737 | <0.0001 |
| Spain-Poland |  | 0.674 | <0.0001 |  | -0.003 | 1.000 |  | 0.622 | <0.0001 |  | 0.160 | <0.0001 |
| Germany-Belgium | cereals or grains regularly * | 0.000 | - | Nuts or seeds regularly * | 0.140 | 0.018 | Olive oil regularly * | 0.116 | 0.369 | Juice and softdrinks > once per week * | 0.008 | 1.000 |
| Italy-Belgium |  | 0.000 | - |  | 0.057 | 1.000 |  | 0.432 | <0.0001 |  | 0.061 | 0.268 |
| Poland-Belgium |  | 0.000 | - |  | 0.063 | 1.000 |  | -0.215 | <0.0001 |  | -0.023 | 1.000 |
| Spain-Belgium |  | 0.000 | - |  | 0.188 | <0.0001 |  | 0.428 | <0.0001 |  | 0.000 | 1.000 |
| Italy-Germany |  | 0.000 | - |  | -0.083 | 0.093 |  | 0.316 | <0.0001 |  | 0.053 | 0.177 |
| Poland-Germany |  | 0.000 | - |  | -0.077 | 0.389 |  | -0.331 | <0.0001 |  | -0.031 | 0.254 |
| Spain-Germany |  | 0.000 | - |  | 0.048 | 1.000 |  | 0.312 | <0.0001 |  | -0.008 | 1.000 |
| Poland-Italy |  | 0.000 | - |  | 0.006 | 1.000 |  | -0.647 | <0.0001 |  | -0.084 | <0.0001 |
| Spain-Italy |  | 0.000 | - |  | 0.131 | <0.0001 |  | -0.004 | 1.000 |  | -0.061 | 0.004 |
| Spain-Poland |  | 0.000 | - |  | 0.125 | 0.002 |  | 0.643 | <0.0001 |  | 0.023 | 0.526 |
| Germany-Belgium | Unsweetened dairy product every day ^+^ | 0.019 | 1.000 | Pastry. sweets. candy every day ^+^ | -0.005 | 1.000 | 2x unsweetened dairy product every day * | -0.048 | 1.000 | 2x pastry. sweets. candy every day * | 0.029 | 1.000 |
| Italy-Belgium |  | 0.038 | 0.115 |  | -0.005 | 1.000 |  | 0.161 | <0.0001 |  | -0.016 | 1.000 |
| Poland-Belgium |  | -0.035 | 1.000 |  | -0.008 | 1.000 |  | -0.208 | 0.001 |  | -0.100 | 0.029 |
| Spain-Belgium |  | 0.038 | 0.305 |  | -0.009 | 1.000 |  | 0.068 | 1.000 |  | 0.012 | 1.000 |
| Italy-Germany |  | 0.020 | 1.000 |  | 0.001 | 1.000 |  | 0.209 | <0.0001 |  | -0.046 | 1.000 |
| Poland-Germany |  | -0.054 | 0.228 |  | -0.003 | 1.000 |  | -0.159 | 0.004 |  | -0.129 | <0.0001 |
| Spain-Germany |  | 0.019 | 1.000 |  | -0.004 | 1.000 |  | 0.117 | 0.038 |  | -0.017 | 1.000 |
| Poland-Italy |  | -0.073 | <0.0001 |  | -0.004 | 1.000 |  | -0.369 | <0.0001 |  | -0.083 | 0.006 |
| Spain-Italy |  | 0.000 | 1.000 |  | -0.005 | 1.000 |  | -0.093 | 0.005 |  | 0.029 | 1.000 |
| Spain-Poland |  | 0.073 | <0.0001 |  | -0.001 | 1.000 |  | 0.276 | <0.0001 |  | 0.112 | <0.0001 |

*Pearson's χ2 test; + Fisher's exact test
